# Implementation of longevity-promoting supplements and medications in public health practice: achievements, challenges and future perspectives

**DOI:** 10.1186/s12967-017-1259-8

**Published:** 2017-07-20

**Authors:** Alexander Vaiserman, Oleh Lushchak

**Affiliations:** 1 Laboratory of Epigenetics, Institute of Gerontology, Vyshgorodskaya St. 67, Kiev, 04114 Ukraine; 2 Department of Biochemistry and Biotechnology, Vasyl Stefanyk Precarpathian National University, Ivano-Frankivsk, Ukraine

**Keywords:** Geroscience, Aging, Healthspan, Longevity dividend, Life extension, Anti-aging drugs

## Abstract

**Background:**

Most modern societies undergo rapid population aging. The rise in life expectancy, nevertheless, is not accompanied, to date, by the same increment of healthspan. Efforts to increase healthspan by means of supplements and pharmaceuticals targeting aging-related pathologies are presently in spotlight of a new branch in geriatric medicine, geroscience, postulating that aging could be manipulated in such a way that will in parallel allow delay the onset of all age-associated chronic disorders.

**Discussion:**

Currently, the concept of the “longevity dividend” has been developed pointed out that the extension of healthspan by slowing the rate of aging is the most efficient way to combat various aging-related chronic illnesses and disabling conditions than combating them one by one, what is the present-day approach in a generally accepted disease-based paradigm. The further elaboration of pharmaceuticals specifically targeted at age-associated disorders (commonly referred to as ‘anti-aging drugs’) is currently one of the most extensively developed fields in modern biogerontology. Some classes of chemically synthesized compounds and nutraceuticals such as calorie restriction mimetics, autophagy inductors, senolytics and others have been identified as having potential for anti-aging intervention through their possible effects on basic processes underlying aging. In modern pharmaceutical industry, development of new classes of anti-aging medicines is apparently one of the most hopeful directions since potential target group may include each adult individual.

**Summary:**

Implementation of the geroscience-based approaches into healthcare policy and practice would increase the ratio of healthy to unhealthy population due to delaying the onset of age-associated chronic pathologies. That might result in decreasing the biological age and increasing the age of disability, thus increasing the age of retirement and enhancing income without raising taxes. Economic, social and ethical aspects of applying the healthspan- and lifespan-promoting interventions, however, have to be comprehensively debated prior to their implementation in public health practice.

## Background

### Population aging: global trends and challenges

Life expectancy has been substantially improved worldwide over the last century. To a large extent, this improvement in longevity is attributable to advances in public health practice, education and medicine [1]. Over the past decades, implementation of vaccination, antibiotics and disinfectants resulted in a significant reduction of infectious diseases as a leading cause of death [2]. Continuing decline in mortality throughout the last decades among the elderly is most likely owing to the widespread implementation of healthy lifestyle behaviors, such as proper diets and exercise, as well as reduction in tobacco smoking [3]. It is generally assumed that if such demographic trend will continue then about 20% of the global population will be older than 60 years by 2050 [4]. Thereby, most of present-day societies undergo rapid population aging. Recently, Bloom et al. [5] estimated that, over the past six decades, the proportion of people aged 60 years and above has increased from 8 to 10%. In the next four decades, however, the faster growth is expected and this group should be increased to 22% of the total world population—from present 800 million to 2 billion people.

The rise of life expectancy, nevertheless, is not accompanied, to date, by the same increase in healthspan [6]. Since aging is the main risk factor for most chronic pathologies, prevalence of age-related diseases, such as type 2 diabetes (T2D), cardiovascular disease (CVD), osteoporosis, neurodegenerative diseases and cancer rises to a large extent with increasing the average lifespan, representing a great socio-economic problem in developed societies. For example, it was recently estimated that more than 30 million people aged over 80 years will be residing in the United States by 2050; about half of these subjects will suffer from dementia and about 3 million will be diagnosed with Parkinson’s disease [7]. The expected increase in prevalence of aging-related pathological conditions will apparently have great impact on economic productivity in many countries during the coming years, including enhancing psychological and financial burden for families and considerable pressure on the government healthcare programs and budgets [5, 8, 9]. Thereby, the development of efficient health interventions, such as disease-prevention and health-promotion programs that target major causes of morbidity in the elderly, might allow minimize the cost pressure related to population aging by providing that the population stays healthy until very old age [10]. The world-wide demographic trend consisting of increasing the proportion of elder persons in populations of various countries could likely explain the dramatic rise in the interest of both general public and medical communities to biogerontology research [11].

The research aimed to promote human longevity understandably raise concern among the general public, as well as among policy makers and government regulators, regarding the growth of the older population and, consequently, higher prevalence of chronic pathologic conditions associated with aging. Experimental studies conducted in various animal models have, however, shown that artificial life extension is generally accompanied by reduced or delayed morbidity including neurodegeneration, CVD, and cancer [12]. For example, dietary restriction was repeatedly shown to be able not only extend the lifespan, but also slow the rate of functional decline and delay the onset of age-related chronic diseases in different model organisms [13]. There is also accumulating epidemiological evidence which is consistent with findings from animal studies. For instance, centenarians, in particular those residing in so-called ‘Blue Zones’ (areas in the US, Latin America, Asia and Europe where unusually many centenarians were revealed), have not only exceptional longevity but also, as a rule, remain free from disabilities and chronic illness to a very old age [14, 15].

### Geroscience: life to years not years to life

Over the past decades, the compression of the morbidity was a basic strategy in gerontology. This strategy is aimed at limiting morbidity to a short time period near the end of life, thereby reducing the burden of diseases and disabilities through delay in the age at onset of the most common aging-related pathological conditions [16]. A few years ago, a new direction in geriatric medicine, geroscience, began to develop. This interdisciplinary field of research is aimed at understanding the mechanistic links between aging and aging-associated diseases [17, 18] and centered primarily on extension of healthspan [19]. According to the “geroscience hypothesis”, aging could be manipulated in such a way that will in parallel allow delay the onset of all age-associated chronic disorders, because these pathologies share the same primary underlying risk factor (age) [13, 17].

Healthspan extension is a central component of activities aimed at achievement of ‘optimal longevity’, a condition defined as ‘living long, but with good health and quality of life’ [16] including improved productivity, functioning and independence. Currently, the research attempted to enhance healthspan are focused primarily on slowing the biological processes underlying aging such as dysfunctions of mitochondria, impaired proteostasis and stem cell function and maintenance, deregulated sensing of cell energy status and growth pathways, cellular senescence, age-related decrease in stress resistance, as well as oxidative and inflammatory stress [20–22]. These processes interact, influencing each other in order to maintain the normal pathways of cellular signaling and to support organismal homeostasis. The compensatory mechanisms mediating these processes, however, became exhausted when reaching a certain age and various aging aspects are manifested, enhancing as a consequence the risk of functional declines and progression of age-associated chronic pathologies [23].

### Anti-aging medicine

The research field targeted at providing therapeutic options to combat the aging-related functional declines and chronic disorders is generally referred to as ‘anti-aging medicine’. This area of investigation, emerging since the beginning of 1990s, has become a hotly discussed topic in the past two decades [24, 25]. Its main purpose is to promote healthspan and lifespan by specific dietary and exercise regimes, as well as by biomedical interventions aimed at delaying or slowing the aging process [26, 27].

Aging is traditionally regarded as ‘natural’ and consequently unpreventable process. However, in the opinion of many field experts, the idea that aging is inevitable part of human nature is rather questionable [28]. Indeed, most present-day evolutionary theories postulate that aging has arisen as a by-product of fundamental evolutionary processes and does not have any specific function [29]. If aging is in fact not an inadmissible component of life, then it might be manipulated like other processes that are commonly believed to be pathological or unnatural. The basic supposition underlying anti-aging research is that age-associated senescence may be regarded as a complex of pathophysiological processes that could be prevented, delayed or even reversed [30]. Currently, biotechnological innovations that potentially may slow down or postpone processes involved in aging are widely implemented in anti-aging medicine [3]. Achievements in this field are to a great extent attributed to an increasingly widespread implementation of ‘omics’ platforms such as genomics, transcriptomics, proteomics and metabolomics [31].

Through the broad implementation of such technologies, a deeper understanding was gained of the fundamental molecular and cellular processes underlying aging, including genomic instability, epigenetic deregulation, loss of proteostasis, mitochondrial dysfunction, cellular senescence, exhaustion of stem cells, inflammation, telomere shortening, autophagy, impaired stress resistance and deregulated nutrient signaling [32–34]. Based on this new knowledge, novel therapeutic strategies to counteract age-associated functional declines and pathological conditions are being developed. In the long run, most promising among them are apparently stem cell- and gene therapy-based approaches. Presently, however, due to insufficient knowledge regarding the potential side effects of these technologies, including cancer, uncertainties and concerns still prevail about their safety among the general public and medical professionals. Therefore, the use of more traditional pharmacological interventions can be considered as a reasonable alternative now [35].

### Anti-aging pharmacology: opportunities and challenges

The further elaboration of pharmaceuticals (both supplements and clinically approved drugs) specifically targeted at age-related pathologies is one of the most rapidly developing fields in modern biogerontology. Over the last 20 years, an exponential rise of research dedicated to investigating substances with potential for use in geriatric practice is observed [36].

In pharmacological research, the first step in the process of drug development is search for druggable molecular targets [37]. In this context, experimental approaches based on using gain- or loss-of-function phenotypes are very helpful to determine gene targets substantially implicated in aging processes [33]. In recent years, this approach has been used to identify many genetic pathways strongly linked to aging and longevity [33, 38]. Nowadays, all these pathways are considered to be promising targets for drugs and several pharmaceuticals targeting them are already under intensive investigation and development.

Identification of processes underlying aging and further development of interventions addressing these processes is apparently a challenging task considering the extreme complexity of aging-related processes. Substantial progress has been, however, achieved during recent years in this field of investigation. Some classes of chemically synthesized compounds and nutraceuticals were identified as having potential for anti-aging intervention [4, 35]. Several substances capable of mimicking the effects of calorie restriction, such as rapamycin, resveratrol and metformin, are thought to be among the most promising in this respect now [39] In addition, high hopes are placed by some authors on antioxidants (coenzyme Q10, quercetin, melatonin, vitamins A, C and E, etc.) [40], inductors of autophagy such as, e.g., spermidine [41], agents that can selectively target and remove senescent cells (senolytics) [42], phytochemicals, such as epigallocatechin gallate (EGCG), catechins, genistein, curcumin, etc. [43], and also several other natural and synthesized compounds. All these substances have been shown to be able to extend lifespan up to 25–30% in different animal models [35]. Over the last years, biotechnological applications are widely used in anti-aging pharmacology. For instance, nanotechnology-based drug delivery systems were recently proposed as a novel promising approach for Alzheimer’s and Parkinson’s diseases treatment [44]. Small size and thus ability to penetrate the blood–brain barrier is the major advantage on these systems. Medications targeted to enzymes participating in processes of epigenetic regulation of gene expression, for example, inhibitors of histone deacetylases such as suberoylanilide hydroxamic acid, trichostatin A and sodium butyrate are another promising drug class for anti-aging intervention [45]. An overview of the most common anti-aging drugs is given in Table 1. Only agents that have reached clinical trials for treating various age-associated clinical conditions are included in the table. The most comprehensive to date overview of the current state-of-the-art in the field of anti-aging pharmacology is provided in recent paper by Vaiserman et al. [35].

**Table 1:** Summary of topical medications with anti-aging properties

Compound (chemical class)	Sources	Medication/supplement name(s)	Effects on organism	Clinical trial phase	Targets	Side effects
Aspirin	Meadow sweet Willow bark Blueberry Broccoli Cauliflower Eggplant Kiwi Grapes	Ecotrin Aspir 81 Aspir-low Aspirin low strength	Anti-inflammation Anti-cancer Anti-stress	Pain reliever/fever reducer (FDA-approved) T2D (phase 3/4) Heart disease (phase 3/4) Atherosclerosis (phase 4) Cancers (phases 1/2/3/4) Obesity (phase 1)	COX-1, COX-2, PTGS2, NF-κB, AMPK	Diarrhea Headache Loss of appetite Vomiting Weight gain
Curcumin (polyphenol)	Curry spice Ginger Turmeric	Theracurmin Meriva Longvida BCM-95	Anti-inflammation Anti-cancer Anti-atherogenic Anti-diabetic Anti-depressant Neuro-protective Anti-stress	GRAS by FDA AD (phase 2) Cancer (phase 2)	NF-κB, COX-1, COX-2, TNF-α, p53, PPARγ, TR, Nrf2, FAK, Src, GSK3, AP1, TOR, LOX, AMPK	Flatulence Nausea Diarrhea
Epigallo-catechin gallate	Green tea Apples Blackberry Carob flour	Green tea extract	Anti-inflammation Anti-cancer Anti-amiloid Anti-atherogenic Anti-obesity Anti-diabetic Neuro-protective Anti-stress	GRAS by FDA AD (phase 2/3)	Bcl2, NOS2, LamR, EGFR, Telomerase, Topoisomerase II, DNMT1	Headache Nervousness Vomiting Diarrhea Irritability Irregular heartbeat Dizziness
Fisetin (flavonoid)	Acacias parrot tree Honey locust onion Strawberry Apple Grapes	Fisetin	Anti-inflammation Anti-cancer Anti-atherogenic Anti-obesity Anti-diabetic Anti-oxidant CR mimetic Anti-stress	Preclinical studies	Akt, Cdk6, mTOR, PI3 K, ERK	Not reported
Melatonin (biogenic amine)	Tomato Cereal Walnut Olive oil Strawberry Milk Wine Beer	Melatonin Circadin Clocktonin	Neuro-protective Anti-stress Anti-migraine Sedation Sleep quality Anti-depressive Anti-stress	GRAS by FDA Cancers (phases 1/2/3/4) Glucose tolerance (phase 3) Insomnia (phase 2) T2D (phase 2) Alzheimer’s disease (phase 2)	MT1, MT2, MT3, GPR50	Headache Depression Sleepiness Dizziness Irritability
Metformin (biguanide)	Chemically synthesized	Act Metformin Bio-metformin Fortamet Glucophage Glumetza Metformin Riomet	Anti-inflammation Anti-cancer Anti-atherogenic Anti-diabetic Anti-depressant Neuro-protective Cardio-protective CR mimetic	T2D (phase 4) Obesity (phase 4) Impaired glucose tolerance (phase 4)	AMPK	Lactic acidosis Diarrhea Nausea Vomiting Flatulence
Quercetin (flavonoid)	Greens Berries Tomato Broccoli Onions Tea leaves	Quercetin	Anti-atherogenic Anti-inflammation Cardio-protective Anti-oxidant	GRAS by FDA T2D (phase 1) CVD (phase 1)	SIRT1, PLA2, PI3K, pp60src Phospho-transferase, Protein kinases, Cyclic GMP phospho-diesterases	Not reported
Resveratrol (polyphenol)	Grapes Wine Raspberry Plums Acai Peanuts	Resveratrol	Anti-inflammation Anti-cancer Anti-atherogenic Anti-obesity Neuro-protective Cardio-protective CR mimetic Anti-stress	GRAS by FDA AD (phase 3) Cognitive impairment (phase 4)	Sirt2, p53 AMPK, PGC1-α	Intestinal upset Nausea
Rapamycin	*Streptomyces hygroscopicus*	Rapamycin Sirolimus Rapamun	Anti-inflammation Anti-cancer Anti-amiloid Anti-atherogenic Neuro-protective Cardio-protective CR mimetic Anti-stress	CVD (phase 3) Tuberous sclerosis (phase 3) Cancers (phases 1/2/3/4)	mTOR	Suppression of immune system Hepatotoxicity
Statins	Oyster Mushrooms Red yeast rice Soy products Grains Cauliflower Onion Apple Orange	Atorvastatin Fluvastatin Lovastatin Pitavastatin Pravastatin Rosuvastatin Simvastatin	Anti-hyperlipidemic Cardio-protective Anti-diabetic Anti-atherogenic Anti-inflammation Anti-Alzheimer’s	Hypercholesterolemia (FDA-approved) CVD (phase 4) Myocardial infarction (phase 4) Sexual dysfunction (phase 4) T2D (phase 4) Schizophrenia (phase 4)	Hydroxy-methyl-glutaryl-CoA reductase	Headache Depression Sleepiness Dizziness Diarrhea Memory loss Diabetes

Sources of information for the table: Drugs.com [46], ClinicalTrials.gov [47], Geroprotectors [48]; Examine.com [49] and US Food and Drug Administration [50]

*AD* Alzheimer’s disease, *CR* calorie restriction, *CVD* cardio-vascular disease, *GRAS* generally recognized as safe, *T2D* type 2 diabetes

An important point is, however, that most substances with potential anti-aging properties are apparently multifunctional and targeted at various molecular pathways that mediate aging. Furthermore, there is only limited evidence to demonstrate overall health benefits of using such substances so far. Findings from epidemiological studies reporting the long-term health impacts of these agents are rather inconsistent. Moreover, evidence from several studies indicates that uncontrolled consumption of some medications considered as potential anti-aging drugs may be useless or even detrimental.

For example, long-term intake of free radical-scavenging antioxidants is considered by most medical professionals as quite reasonable option to promote health and wellbeing, and also to prevent various aging-related conditions such as atherosclerosis, inflammatory disorders, CVD and cancer [51]. The health outcomes of these interventions, however, are still under debates. Doubts on this matter are strengthened by the results obtained in a series of meta-analyses of randomized controlled trials and observational studies by Bjelakovic and co-authors. Based on these meta-analyses, the authors concluded that long-term intake of dietary antioxidants, such as beta-carotene and vitamins A and E, can be associated with unfavorable health outcomes and with increased cancer and all-cause mortality, especially in well-nourished populations [52, 53]. One more example of potential risk of using compounds with anti-aging properties, such as e.g. calorie restriction mimetics, is that they might induce insulin resistance. Such side effects were observed, in particular, in patients treated with rapamycin [54]. Treatment with rapamycin, among other mechanistic Target of Rapamycin (mTOR) inhibitors, was associated with a 13–50% higher incidence of hyperglycemia and development of T2D if applied as anticancer therapies [55]. Similarly, insulin resistance-inducing effects were also reported for statins. The risk for development of T2D was found to be increased by 9–12% in two meta-analyses of statin trials and by 18–99% in five population-based studies [56].

Another reasonable approach in anti-aging pharmacology is evaluation of the geroprotective potential of medications already approved by the US Food and Drug Administration (FDA) and other regulatory authorities for treating various pathological conditions related to aging. Among them, metformin, statins, beta-blockers, thiazolidinediones, newer generation β-adrenergic receptor inhibitors, renin-angiotensin-aldosterone system inhibitors, as well as anti-inflammatory medications appear to be the most promising drug candidates in this respect [16]. The safety of these drugs has been confirmed in a number of clinical trials. This is also compelling evidence that they may improve health, well-being and physiological functioning in elderly patients suffering from chronic pathologies [57]. One problem is that these substances are not used currently for treating age-related pathological conditions in the absence of clinical manifestations of particular illness. There are, however, good reasons to suggest that these agents could theoretically be redirected to preventing or treating other syndromes or conditions commonly associated with aging.

### Longevity dividend

Despite an extraordinary rapid technological progress in pharmacology, there are few new preparations in the development pipeline now. Thereby, drugs generated on the basis of new knowledge gained from biogerontological research that can delay or prevent most age-associated disorders would apparently become “blockbusters” of modern pharmaceutical industry and market [58]. That follows the idea that the extension of the healthy life expectancy by slowing aging process is the most efficient way to combat aging-related chronic illnesses and disabling conditions representing serious medical, social and economic issue in modern societies. This idea is referred to as the “longevity dividend” in the contemporary literature [59].

Discovery and development of anti-aging drugs could likely provide an opportunity for revitalization of the drug development pipeline [58]. Indeed, if it would be possible to slow down the aging process per se, then that would allow delay or prevent most aging-related disorders rather than combating them one by one, which is the conventional approach in the present-day disease-based paradigm of drug development. Moreover, population life expectancy might be only insignificantly affected by preventing only certain pathological conditions. This is due to the fact that other disorders will, due to comorbidity, greatly devalue the positive effects achieved by prevention of the targeted disease. Therefore, simultaneous delaying the clinical manifestations of all aging-associated disorders by inhibiting fundamental aging mechanisms may be much more effective than prevention of particular chronic diseases [16, 57]. It can be also assumed that not only considerable health advantages but also substantially larger socio-economic benefits may be achieved from such approach relative to the conventional approach in present-day public health practice targeted to preventing certain pathological conditions only [60]. Goldman et al. have estimated the economic advantages from enhancing healthspan through slowing down aging process in the US at about 7 trillion dollars over the next 50 years [61].

### Prospect debate: should aging be recognized as a disease?

Until recently, substances with potent anti-aging properties were not considered by US FDA and other regulatory agencies as suitable candidate drugs for clinical trials, because they did not recognize aging as a clinical condition. However, it has become increasingly apparent now that such opinion is rather contradictory. Indeed, it’s obvious that aging is generally accompanied by health issues typically associated with clinical conditions such as loss of muscle mass (sarcopenia), osteoporosis, atherosclerosis, hypertension, strokes, heart attacks and atrophy of brain tissues resulting in dementia. Clinically, all of these conditions are recognized as diseases and require specific therapeutic interventions. Therefore, an intense debate has emerged now in both academic and policy making circles on whether or not aging can be classified as a disease [62, 63]. Perhaps as a consequence of this discussion, the FDA’s position on that occasion has become a lot less rigorous in recent years.

### Anti-aging pharmacology: an FDA perspective

The first example of this ‘paradigm shift’ is approval by FDA of clinical trial for determining the efficacy of the anti-diabetic drug, metformin, in reducing the risk for aging-associated diseases including CVD, cognitive impairment and cancer, in non-diabetic patients. This substance has been selected for clinical trial because it was previously demonstrated to affect different pathways implicated in aging process such as oxidative damage, inflammation, cellular senescence, apoptosis and autophagy. Patients with T2D treated with metformin also had longer survival time than did age- and gender-matched, non-diabetic controls [64]. In this clinical trial named TAME (Targeting Aging with MEtformin), 3000 volunteers aged 70–80 years will be treated with metformin for 5–7 years to determine whether such treatment would be effective in preventing or delaying the onset of age-associated pathologies [22, 65]. Such decision could be indicative of the change of FDA’s position on the anti-aging pharmacology from regulations for cosmetic manufacturers to regulations for prevention and/or treatment of chronic diseases related to aging [66]. It could establish innovative regulatory pathways for clinical trials of medications designed to slow down the aging process. In addition, recognition of aging as disease would motivate both individual donors and research funding agencies to address more resources to research in the field of biogerontology and, in particular, to developing drugs specifically targeting the aging processes.

## Conclusions

On the basis of the above considerations, it can be suggested that targeting the aging process per se may be a far more effective approach to prevent or delay aging-associated pathologies than treatments specifically targeted to particular clinical conditions. Due to demographic processes linked to population aging, such strategy seems very relevant in the context of modern public health practices [67]. In modern pharmaceutical industry, further development of anti-aging medications is apparently one of the most promising directions since a potential target group may include each adult individual. Some supplements are already promoted as ‘anti-aging pills’ in modern pharmaceutical market. The most widespread among them is resveratrol, a polyphenol found in grapes and in several other plant sources and repeatedly demonstrated an ability to extend healthspan in various animal models [68].

Marketing research conducted in recent years indicated that most people would like to purchase supplements and medications aimed at delaying or preventing age-associated declines in mental and physical functioning [58]. According to the latest sociological surveys, there is a great desire for extension of human healthspan and lifespan worldwide. Previously, most questionings were based on the erroneous presupposition that life extension can be achieved due to prolongation of the period of functional impairment, frailty and disability at the end of life. As a consequence, it is not surprising that careful attitude to life extension has been revealed in these surveys. When the extended healthspan was postulated in questionnaire design, responses largely favored longer life. For example, in a recent survey by Donner et al. 20% of respondents wanted to live to the age of 85, while 42% wanted that their life expectancy would not be limited [69].

The implementation of the geroscience-based approaches into healthcare policy and practice would increase the ratio of healthy to unhealthy population due to delaying the onset of aging-associated chronic pathologies. In other words, that might result in decreasing the biological age (i.e., elder subjects will become biologically younger) and increasing the age of disability, thus increasing the age of retirement and income without raising taxes [70, 71]. Social, economic and ethical aspects of applying the healthspan- and lifespan-extending interventions should however be comprehensively debated prior to their implementation in public health practice.

## Abbreviations

AD: Alzheimer’s disease
CR: calorie restriction
CVD: cardio-vascular disease
FDA: Food and Drug Administration
mTOR: mechanistic target of rapamycin
TAME: Targeting Aging with MEtformin
T2D: type 2 diabetes
